# Mercury exposure, epigenetic modifications, and genetic susceptibility: insights from molecular docking and population analysis

**DOI:** 10.3389/fpubh.2025.1710032

**Published:** 2025-11-17

**Authors:** Bakhtiyar Serik, Lyazzat E. Shinetova, Natalya V. Efimova, Saulemay A. Bekeyeva, Balkiya M. Abdrakhmanova, Aliya O. Dauletova, Roza K. Suleimenova, Nadiar M. Mussin, Afshin Zare, Ramazon Safarzoda Sharoffidin, Amin Tamadon

**Affiliations:** 1 Medical School, M. Kozybayev North Kazakhstan University, Petropavlovsk, Kazakhstan; 2 Psychometric Laboratory, National Testing Center, Astana, Kazakhstan; 3 East Siberian Institute of Medical and Ecological Research, Angarsk, Russia; 4 Department of Epidemiology and Biostatistics, Astana Medical University, Astana, Kazakhstan; 5 Department of Medical Genetics and Molecular Biology, Astana Medical University, Astana, Kazakhstan; 6 Department of General Surgery, West Kazakhstan Marat Ospanov Medical University, Aktobe, Kazakhstan; 7 College of Medicine, Taipei Medical University, Taipei, Taiwan; 8 Department of Pharmaceutical Technology, Avicenna Tajik State Medical University, Dushanbe, Tajikistan; 9 Department of Natural Sciences, West Kazakhstan Marat Ospanov Medical University, Aktobe, Kazakhstan

**Keywords:** mercury, environmental exposure, genetic polymorphism, glutathione transferase, toxicokinetics, toxicodynamics, public health, mining activities

## Abstract

**Introduction:**

Mercury (Hg) is a major environmental contaminant and public health concern, particularly in industrial regions where metallurgical activities contribute to elevated Hg emissions. Genetic factors influencing susceptibility to mercury toxicity remain underexplored in Central Asia. This study investigated genetic predisposition to Hg accumulation and toxicity among residents of Temirtau, Kazakhstan.

**Methods:**

A total of 180 residents from Temirtau and 90 control participants were enrolled. Mercury concentrations were measured in blood and hair samples using cold vapor atomic absorption spectrometry. Dietary information was collected to identify major exposure routes. Genotyping for GSTM1, GSTT1, GSTP1 (Ile105Val, rs1695), and GCLM (–588C/T, rs41303970) polymorphisms was performed using PCR and PCR-RFLP methods. Complementary molecular docking analyses were conducted to assess methylmercury (MeHg) interactions with key epigenetic regulators—DNA methyltransferase 1 (DNMT1), histone deacetylases (HDAC1–6), and sirtuin 1 (SIRT1).

**Results:**

Individuals carrying GSTM1-null and GCLM variant genotypes exhibited higher Hg accumulation and greater oxidative-stress susceptibility compared with wild-type carriers. Molecular docking revealed moderate binding affinity of MeHg within the catalytic domains of DNMT1 and HDAC isoforms, suggesting interference with DNA methylation and histone-modification processes. Although exposure levels were considerably lower than those in classical Minamata incidents, subclinical effects and genotype–environment interactions were evident.

**Discussion:**

These findings highlight oxidative stress and epigenetic dysregulation as potential mechanisms underlying interindividual variability in mercury toxicity. The integration of genetic and molecular-modeling approaches provides valuable insights for risk assessment and preventive strategies in populations chronically exposed to industrial pollutants.

## 1 Introduction

Mercury (Hg) is a toxic heavy metal that threatens human health and the environment (1). Hg exists in elemental, inorganic, and organic forms and enters ecosystems through both natural processes and human activities such as mining and metallurgical emissions (2). When released, Hg is altered into methyl mercury (MeHg), the most toxic form, through a process called Meation, in biological organisms, especially humans (3).

MeHg is highly bioavailable which tends to bioaccumulate in biological tissues. For instance, it remains in the bloodstream for a long time and is considered a serious health problem for certain high-risk groups (2), particularly during pregnancy or childhood. Neurological disorders as a result of MeHg exposure include visual impairments, ataxia, cognitive deficits and severe developmental disabilities in children (4). The transplacental transmission of MeHg from mother to fetus is especially concerning, as it can adversely affect fetal development, leading to devastating outcomes later in life (5).

Biological markers such as blood, urine, nails, and hair are widely used to assess Hg exposure (6). Hair analysis, in particular, reflects chronic MeHg exposure linked to fish and shellfish consumption (7, 8). Although dietary intake is the dominant global pathway (>98% of total exposure) (9), in Temirtau, Kazakhstan, industrial emissions from metallurgical and chlor-alkali plants constitute the primary exposure source, with local fish consumption serving as a secondary route via aquatic bioaccumulation. Monitoring Hg levels in biological samples is therefore essential for understanding both individual and population exposure.

Food consumption accounts for the majority (>98.85 to 99.48%) of Hg exposure in most global populations, according to a report by the Korea Food and Drug Administration (KFDA) (9), while pathways other than food consumption contribute much less. However, the exposure profile in Temirtau differs markedly: in this industrial city, emissions from metallurgical and chlor-alkali plants represent the primary source of Hg release into the environment, contaminating air, soil, and the nearby Nura River system. Fish consumption serves as a secondary exposure route, reflecting local bioaccumulation of mercury within aquatic food webs. Additionally, the types of biomarkers—exposure, effect, and susceptibility—illustrate the multifactorial nature of assessing the health effects of Hg (6). Compared to blood and urine concentrations that reflect recent exposure, hair provides a longer-term indicator of chronic exposure (10). Therefore, monitoring and determining Hg concentrations in these biological samples is essential for understanding both individual and population exposure to Hg and associated health effects.

We selected residents of Temirtau for this study because of their long-term and current exposures to Hg from industrial emissions and associated environmental contamination, as highlighted in the introduction section related to the study population. This setting presents a unique opportunity to study genetic variability in response to Hg exposure in a community frequently neglected in discussions of environmental health hazards. In Temirtau, industrial activities—particularly metallurgical and chemical production—have led to measurable Hg contamination in local ecosystems, with air, soil, and water pathways contributing to chronic exposure. Hair Hg levels in residents averaged 2.1 μg/g (range: 0.5–5.8 μg/g), exceeding the WHO safety threshold (1 μg/g) (11). Blood Hg concentrations in the study cohort mirrored these findings, with a median of 8.7 μg/L, consistent with chronic low-to-moderate exposure (12). Epidemiological surveys in the region report a 12–18% prevalence of mild neurocognitive symptoms among adults with hair Hg > 2 μg/g, aligning with global patterns in industrially exposed communities (13). Similar industrial regions, such as Minamata (Japan) (14) and Angarsk (Russia) (15), demonstrate comparable exposure-dose relationships, with neurodevelopmental effects observed at hair Hg levels >1.5 μg/g. Although local fish consumption (27 kg/year per capita) (16) contributes additional exposure through bioaccumulated methylmercury, it remains a secondary pathway compared with industrial emissions. While direct biomonitoring data for Temirtau remain limited, extrapolation from regional industrial emissions and dietary surveys suggests elevated exposure risks, necessitating this genetic susceptibility study. Temirtau, located in central Kazakhstan (Karagandy region), is an industrial city historically associated with metallurgical production and chlor-alkali plants releasing mercury into the Nura River basin. Previous monitoring reported environmental Hg levels up to 0.3–1.2 mg/kg in sediments and elevated human biomarker values.

Glutathione S-transferase Mu 1 (*GSTM1*), glutathione S-transferase theta-1 (*GSTT1*), glutathione S-transferase pi 1 (*GSTP1*), and glutamate-cysteine ligase modifier subunit (*GCLM*) were selected for this study because of their important roles in the detoxification of xenobiotics (including heavy metals such as Hg) (17). These genes encode glutathione S-transferases, which are enzymes that catalyse the conjugation of glutathione to toxic compounds, thus helping to remove them from the body (18). Genetic polymorphisms of these genes can affect the enzymatic activity and, thereby, the individual’s ability to detoxify Hg (19). Knowledge of genetic differences in metabolism of Hg in these pathways may help to explain individual susceptibility to Hg in a population exposed to high levels of environmental pollutants.

The genes *GSTM1*, *GSTT1*, *GSTP1*, and *GCLM* were selected based on their well-established roles in Hg detoxification and oxidative stress response. *GSTM1* and *GSTT1* encode glutathione S-transferases that conjugate glutathione to MeHg, facilitating its excretion; deletions in these genes (*GSTM1*/*GSTT1*-null) impair detoxification and increase susceptibility to Hg toxicity (17, 19). *GSTP1* (Ile105Val, rs1695) alters enzyme affinity for Hg conjugates, while *GCLM* (–588C/T, rs41303970) modulates glutathione synthesis, a critical antioxidant defense against Hg-induced oxidative damage (18, 20). These polymorphisms were prioritized due to their high prevalence in global populations and documented associations with Hg-related neurotoxicity in industrial cohorts (12, 21).

This study aimed to determine the relationship between Hg exposure levels and the frequency of GSTM1, GSTT1, GSTP1, and GCLM polymorphisms among residents of Temirtau, and to clarify the genetic basis of individual susceptibility to mercury toxicity. To complement the population analysis, we employed molecular docking to investigate interactions between MeHg and key detoxification and epigenetic regulatory proteins (DNMT1, HDACs, and SIRT1), providing a mechanistic framework linking mercury exposure to potential gene expression changes.

## 2 Materials and methods

### 2.1 Study population and sample collection

The sample size was 180 people forming the primary and control group: the first group is the people who live in the Temirtau region – 90 people, and the control group of 90 people Table of Study Samples. Genomic DNA was isolated from venous blood using a sorbent method and conditions similar to the Wizard^®^ Genomic DNA Purification kit by Promega. The control group comprised 90 age- and sex-matched individuals from non-industrial regions of Kazakhstan (specifically, the rural districts of Akmola and Karagandy), with no history of occupational Hg exposure or frequent fish consumption (>1 meal/week). Controls were selected from regions with negligible industrial Hg emissions (<0.1 μg/m^3^ annual average) to ensure contrast with Temirtau’s exposure profile (Table 1). Detailed demographic and clinical characteristics, including smoking, alcohol consumption, fatigue complaints, and genotype combinations, are presented in Table 1. Hg levels were quantified in urine and hair samples using Cold-Vapor Atomic Absorption Spectrometry (CVAAS; Lumex RA-915 M, Russia) with a detection limit of 0.1 μg/L. Analytical precision was verified with standard reference material 1641d (NIST). Mean urinary Hg concentrations were 1.8 ± 1.2 μg/L for residents and 0.4 ± 0.3 μg/L for controls (*p* < 0.001).

**Table 1:** Demographic and clinical characteristics of Temirtau residents vs. controls.

Variable	Temirtau residents (*n* = 90)	Controls (*n* = 90)	Notes
Age (mean ± SD)	64.2 ± 10.5 years	59.6 ± 12.3 years	Controls slightly younger; both groups predominantly middle-aged/older adults
Sex (female:male)	52:38	54:36	Comparable gender distribution.
Nationality	48 Kazakh, 42 Russian	90 Kazakh	Controls exclusively Kazakh; Temirtau group mixed.
Residence Duration	>20 years (all)	>15 years (all)	Both groups long-term residents.
Smoking status	12 current smokers	0 (excluded)	Smoking exclusion criterion for controls.
Chronic diseases	90% with arterial hypertension (АH)	6 with АH (excluded)	Controls excluded for chronic diseases; Temirtau group high comorbidity.
Hg Exposure (μg/L)	1.8 ± 1.2 (urine)	0.4 ± 0.3 (urine)	Higher exposure in Temirtau residents.
Genotype (*GSTP1*)	56 Ile/Ile, 21 Ile/val	53 Ile/Ile, 17 val	Similar genotype distribution; slight variation in heterozygotes.
Occupational hazards	28 miners/drivers	0 (excluded)	Controls excluded for chemical/industrial exposure.

SD = standard deviation; AH = arterial hypertension; μg/L = micrograms per liter.

Dietary Hg exposure was assessed using a semi-quantitative Food Frequency Questionnaire (FFQ) adapted from the WHO 2008 guidance, focusing on weekly fish and seafood consumption, portion size, and cooking method. The FFQ was administered by trained interviewers.

Inclusion criteria: adults (>18 years) residing ≥20 years in Temirtau or control area, without occupational exposure outside the region. Exclusion criteria: pregnancy, neurological disorders, recent chelation therapy, or occupational heavy metal exposure. The study protocol was approved by the Local Bioethics Committee of NCJSC MUA (Approval No. 1, dated 23 Feb 2023).

Mercury concentrations were determined in blood and hair samples using Cold-Vapor Atomic Absorption Spectrometry (CVAAS; Lumex RA-915 M, Russia) with a detection limit of 0.1 μg/L. Calibration was verified with NIST SRM 1641d. Genotyping of GSTP1 (rs1695) and GCLM (rs41303970) polymorphisms was carried out by PCR-restriction fragment length polymorphism (PCR-RFLP), while GSTM1 and GSTT1 deletions were analyzed by multiplex PCR using CYP1A1 as internal control.

### 2.2 DNA quantification

Genomic DNA was extracted from venous blood using the Wizard^®^ Genomic DNA Purification Kit (Promega, United States) according to the manufacturer’s instructions. Concentration and purity were assessed spectrophotometrically (NanoDrop 1,000, Thermo Fisher) with A260/A280 ratios of 1.8 ± 0.1 (mean ± SD), confirming minimal protein contamination. DNA yields ranged from 10–130 ng/μL. Integrity was further verified by electrophoresis on 0.9% agarose gels stained with ethidium bromide, showing intact bands >10 kb without smearing.

### 2.3 Polymerase chain reaction (PCR)

All PCR runs included both negative controls (nuclease-free water instead of DNA template) and positive controls (DNA samples with pre-confirmed genotypes).

#### 2.3.1 *GCLM* gene polymorphism (–588C/T, rs41303970)

Genotyping of the *GCLM* gene polymorphism (–588C/T, rs41303970) was performed using PCR-restriction fragment length polymorphism (PCR-RFLP). The amplification was carried out using the following primers: forward 5′-TTCACGTGCTCACTGGAGTT-3′ and reverse 5′-CAGGGACAGGGAAAAGAGTG-3′, yielding a 310 bp amplicon. Each 30 μL PCR reaction contained 200 ng of genomic DNA, 1 × PCR buffer (10 mM Tris–HCl, 50 mM KCl), 2.5 mM MgCl₂, 0.2 mM dNTPs, 1.5 μL of DMSO, 10 pmol of each primer, and 1 U of Taq DNA polymerase (Fermentas). The PCR conditions included an initial denaturation at 95 °C for 4 min, followed by 35 cycles of denaturation at 95 °C for 30 s, annealing at 55 °C for 40 s, and extension at 72 °C for 40 s. A final elongation step was conducted at 72 °C for 10 min. The PCR products were digested with 5 U of BsaAI restriction enzyme (Thermo Fisher Scientific) in 1 × CutSmart Buffer at 37 °C for 2 h. Genotypes were determined based on the banding patterns observed after electrophoresis on a 2% agarose gel run at 100 V for 45 min: CC genotype yielded fragments of 200 bp, 84 bp, and 45 bp; CT genotype showed 200 bp, 129 bp, 84 bp, and 45 bp; and TT genotype showed 200 bp and 129 bp fragments.

#### 2.3.2 GSTP1 gene polymorphism (exon 5, Ile105Val, rs1695)

The *GSTP1* gene polymorphism (Ile105Val, rs1695) was also genotyped by PCR-RFLP. Amplification was conducted using the primers: forward 5′-GTAGTTTGCCCAAGGTCAAG-3′ and reverse 5′-AGCCACCTGAGGGTAAG-3′, producing a 176 bp amplicon. The PCR reaction mixture was identical to that described in Section 2.3.1, with the exception of the annealing temperature, which was set at 59 °C. The PCR cycling program included an initial denaturation at 94 °C for 3 min, followed by 35 cycles of 94 °C for 40 s, 59 °C for 40 s, and 72 °C for 60 s, with a final extension at 72 °C for 10 min. The amplified product was digested using 5 U of Alw26I enzyme (Thermo Fisher Scientific) in 1 × Buffer R at 37 °C for 3 h. Electrophoresis was performed on a 3% agarose gel at 100 V for 60 min. The genotypes were identified as follows: the Ile/Ile genotype produced a single uncut 176 bp band; the Ile/Val genotype showed three bands of 176 bp, 91 bp, and 85 bp; and the Val/Val genotype exhibited 91 bp and 85 bp fragments.

### 2.4 Multiplex PCR for GSTM1 and GSTT1 gene deletions

A multiplex PCR assay was used to detect deletions in the *GSTM1* and *GSTT1* genes, with co-amplification of the CYP1A1 gene serving as an internal control. The primers used for *GSTM1* were forward 5′-GAACTCCCTGAAAAGCTAAAGC-3′ and reverse 5′-GTTGGGCTCAAATATACGGTGG-3′ (amplicon size: 215 bp); for *GSTT1*, forward 5′-TTCCTTACTGGTCCTCACATCTC-3′ and reverse 5′-TCACCGGATCATGGCCAGCA-3′ (amplicon size: 480 bp); and for CYP1A1, forward 5′-GAACTGCCACTTCAGCTGTCT-3′ and reverse 5′-CAGCTGCATTTGGAAGTGCTC-3′ (amplicon size: 312 bp). Each 30 μL reaction contained 200 ng of genomic DNA, 1 × PCR buffer, 2.0 mM MgCl₂, 0.2 mM dNTPs, 0.5 μM of each primer, and 1 U of Taq DNA polymerase. PCR cycling conditions included an initial denaturation at 95 °C for 5 min, followed by 35 cycles at 95 °C for 30 s, 60 °C for 40 s, and 72 °C for 50 s, with a final extension step at 72 °C for 7 min. PCR products were analyzed by electrophoresis on a 2% agarose gel at 100 V for 60 min. The presence of *GSTM1* and *GSTT1* was indicated by the appearance of 215 bp and 480 bp bands, respectively. Absence of these bands indicated the null genotype for the respective gene. The 312 bp CYP1A1 product was required in all samples to confirm successful amplification.

### 2.5 Protein selection for molecular docking analysis

Proteins selected for molecular docking included DNA Metransferase 1 (DNMT1), histone deacetylases (HDAC1, HDAC2, HDAC3, HDAC6), and sirtuin 1 (SIRT1), based on their well-established roles in epigenetic regulation and their relevance to Hg-induced toxicity. Although glutathione S-transferases (GSTs)—such as *GSTM1*, *GSTT1*, and *GSTP1*—play a central role in detoxification and Hg metabolism, they were excluded from the analysis due to limited availability of high-resolution structural data on human isoforms complexed with Hg species. Instead, the study focused on epigenetic regulators, given that MeHg has been shown to interfere with DNA Meation through DNMT1 inhibition and to disrupt histone acetylation via modulation of HDACs and SIRT1, thereby contributing to long-term neurotoxicity (19, 20).

DNMT1 (PDB ID: 4WXX) was selected due to its critical role in maintaining DNA Meation patterns; Hg-induced dysregulation of DNMT1 is associated with aberrant gene silencing in neurological disorders (2). Multiple HDAC isoforms were included—HDAC1 (PDB: 6Z2J), HDAC2 (PDB: 7ZZO), HDAC3 (PDB: 4A69), and HDAC6 (PDB: 3C5K)—because they are key mediators of chromatin remodeling and gene expression. Previous studies have reported that Hg exposure alters HDAC activity, leading to downstream oxidative stress responses (22). SIRT1 (PDB: 4I5I), a NAD^+^-dependent deacetylase involved in cellular stress response and neuroprotection, was also included, as its inhibition by Hg may exacerbate neurotoxic outcomes (20).

The inclusion of these proteins was further justified by their structural feasibility for docking simulations. Only proteins with available and well-resolved three-dimensional structures in the Protein Data Bank (PDB) were selected to ensure docking reliability and precision. Target sites were chosen based on known thiol-rich domains, such as zinc-finger motifs in HDACs, given Hg’s strong affinity for sulfur-containing residues like cysteine (6). MeHg has been reported to interact with such residues (e.g., CYS279 in HDAC3), potentially altering enzymatic activity (23). Moreover, evidence from Hg-exposed populations suggests that DNMT1 and HDAC expression levels are significantly affected, supporting the relevance of these targets for mechanistic investigation via molecular docking (13) (Table 2).

**Table 2:** Targeted proteins that have been chosen to undergo molecular docking analysis and their PDB ID.

Protein name	PDB ID
DNMT1	4WXX
HDAC1	6Z2J
HDAC2	7ZZO
HDAC3	4A69
HDAC6	3C5K
SIRT1	4I5I

DNMT1 = DNA (cytosine-5)-Metransferase 1; HDAC = histone deacetylase; SIRT1 = sirtuin 1; PDB = Protein Data Bank.

### 2.6 Statistical analysis

Allele and genotype frequencies were compared using chi-square (χ^2^) or Fisher’s exact tests, and odds ratios (OR) with 95% confidence intervals (CI) were calculated to estimate associations between genotypes and mercury exposure. The Hardy–Weinberg equilibrium was tested for all polymorphisms. Bonferroni correction was applied (adjusted *p* < 0.0125).

## 3 Results

Isolation and concentration of DNAIsolated DNA was acquired from 180 samples using a NanoDrop ranging from 10 to 130 ng/μL.

### 3.1 *GCLM* gene

**Figure 1:**
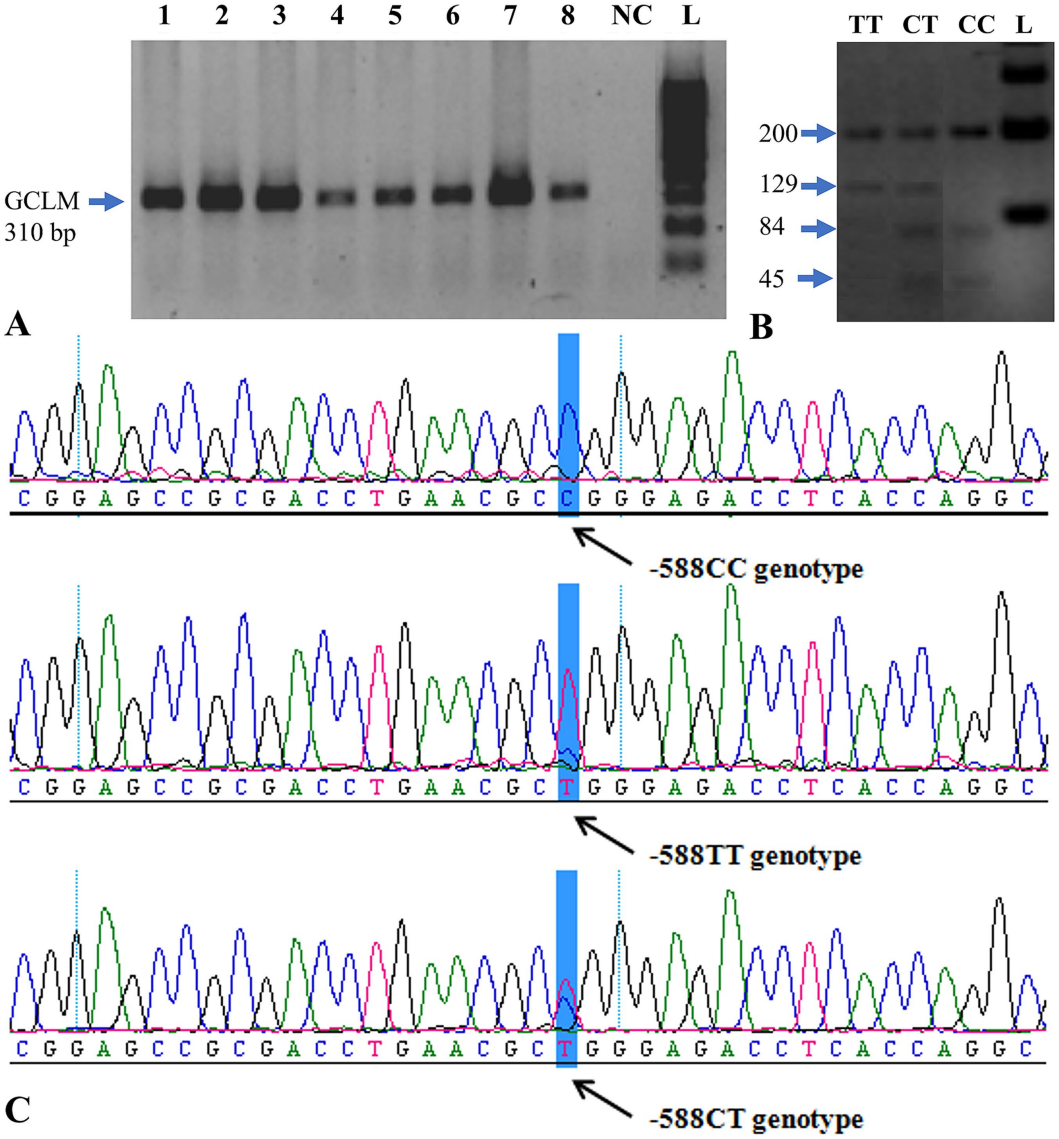
PCR analysis and genotyping of the *GCLM* gene. **(A)** Electropherogram showing successful PCR amplification of the *GCLM* –588C/T region. Lanes 1–8 correspond to representative samples; L = O’GeneRuler^™^ 100 bp Plus DNA Ladder (Fermentas); NC = negative control. **(B)** Agarose-gel electrophoresis of restriction digestion products showing the three observed genotypes: CC (200, 84, 45 bp), CT (200, 129, 84, 45 bp), and TT (200, 129 bp). **(C)** Representative sequencing chromatogram confirming the –588C/T polymorphic site (arrow). PCR = polymerase chain reaction; bp = base pair.

An optimal PCR protocol was developed using primers for the *GCLM* gene to detect the –588C/T polymorphism. The protocol was optimized by adjusting magnesium concentrations. Following this optimization, PCR was conducted on all 180 samples, producing a 310 bp PCR product (Figure 1A).

*GCLM* genotyping was performed following restriction digestion of the *GCLM* gene region. The CC genotype was identified by the presence of 200-, 84-, and 45-bp fragments on gel electrophoresis. The 200-, 129-, 84-, and 45-bp bands were detected in CT genotype samples. TT genotype carriers (200- and 129-bp fragments) were detected in the samples (Figure 1B).

After digestion of exon 5 of the *GCLM* gene with two restriction enzymes, samples with the CC genotype were distinguished by the appearance of 200-, 84-, and 45-bp fragments on agarose gel. The 200-, 129-, 84-, and 45-bp fragments were found in samples with CT genotype. The samples with 200- and 129-bp fragments were found to be TT genotype carriers (Figure 1B).

Without standard PCR buffer (10 mM Tris–HCl, pH 8.3, 50 mM KCl, 1.5 mM MgCl₂) dissolution, the samples were precipitated before the restriction digestion. Twenty-four (24) of the 180 samples were found to be of the CT genotype (sample numbers: 28, 56, 59, 67, 91, 120, 123, 131, 138, 140, 141, 143, 146, 148, 149, 150, 153, 154, 157, 166, 167, 172, 173, 178 and 180). Only the TT allele was seen in the samples 2, 135, 144 and 152. All CT and TT genotype samples as well as 3 control samples were sequenced for validation of the restriction results.

The amplicons were sequenced, and the sequences were analyzed, confirming the existence of the –588C/T polymorphism (Figure 1C). The genotyping confirmed the CT/TT genotypes identified in Table 3 partially. Of the 31 samples sequenced, only 9 gave the same result as that obtained following restriction digestion. Discordant results between PCR-RFLP and sequencing (9/31 samples) likely resulted from partial restriction digestion or PCR artifacts. All discordant samples were re-analyzed to confirm genotypes.

**Table 3:** Restriction and genotyping of the *GCLM* gene region: comparison of results.

No.	Restriction results			Genotyping results		
	CC	CT	TT	CC	CT	TT
1	+			+		
2	+					
3	+					
28		+			+	
56		+		+		
59		+		+		
67		+		+		
91		+		+		
120		+		+		
123		+		+		
131		+		+		
135			+	+		
138		+		+		
140		+				+
141		+				
143		+			+	
144			+			+
146		+		+		
148		+		+		
149	+			+		
150		+		+		
152			+	+		
153	+				+	
154	+			+		
157	+			+		
166	+				+	
167		+			+	
172	+			+		
173	+			+		
178		+		+		
180		+		+		

PCR-RFLP = polymerase chain reaction-restriction fragment length polymorphism; bp = base pair.

Statistical comparison revealed that the GSTT1-null genotype was significantly more prevalent in exposed individuals (OR = 2.91, 95% CI: 1.47–5.78, *p* = 0.001). No significant deviations from Hardy–Weinberg equilibrium were observed for any locus.

### 3.2 Multiplex PCR of GSTM1 and *GSTT1* genes

**Figure 2:**
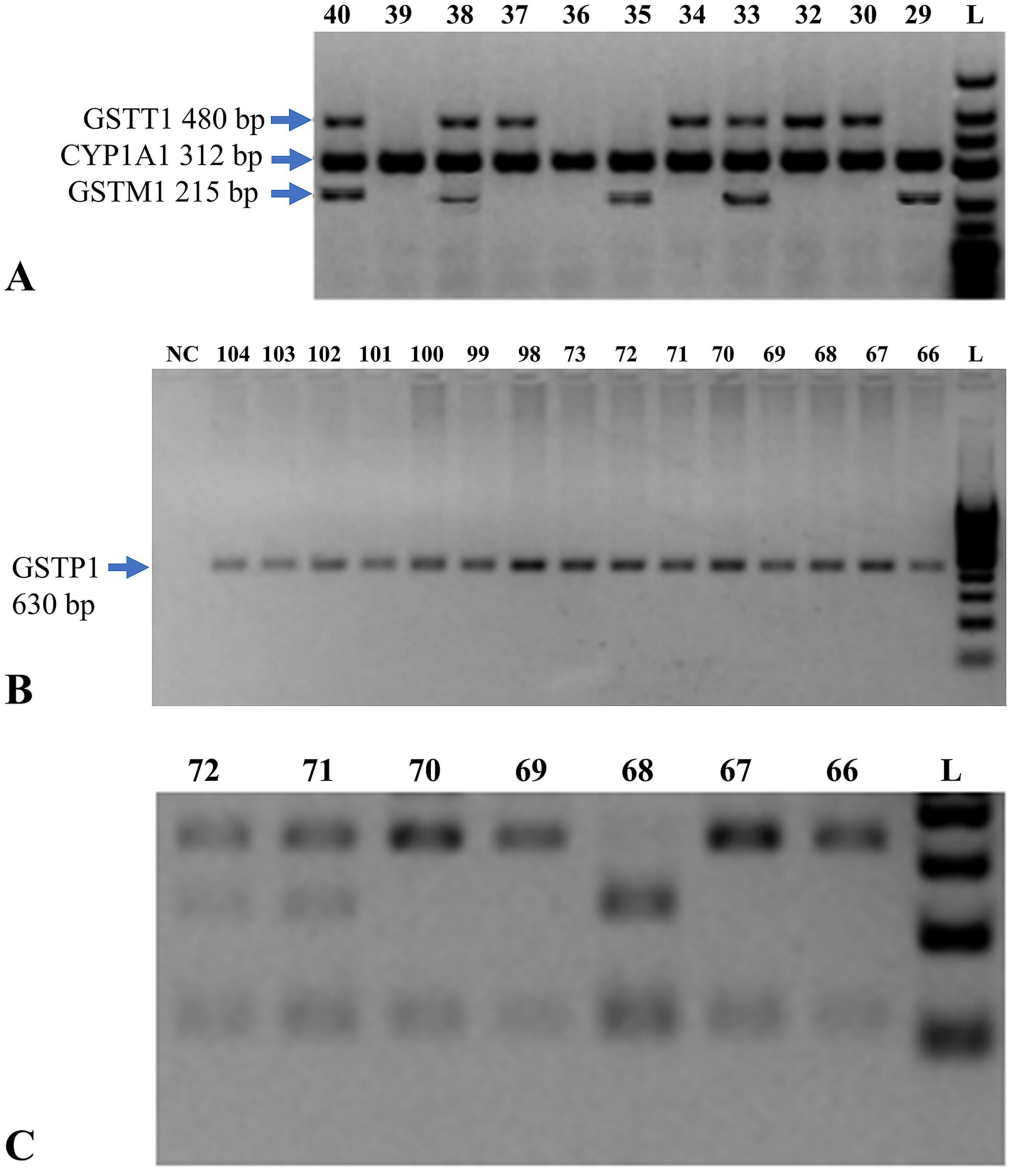
Genetic analysis of *GSTM1*, *GSTT1*, and *GSTP1* genes. **(A)** Multiplex PCR analysis on a 2% agarose gel for detection of *GSTM1* and *GSTT1* genes. The presence of a 215 bp band indicates the *GSTM1* gene, while a 480 bp band indicates the *GSTT1* gene. L = DNA size marker (O’GeneRuler^™^ 100 bp Plus DNA Ladder, Fermentas). **(B)** Electropherogram of PCR amplification of exon 5 of the *GSTP1* gene. Lanes 66–104 represent test samples. L indicates the molecular weight marker (O’GeneRuler^™^ 100 bp Plus DNA Ladder, Fermentas), with fragment sizes ranging from 100 to 1,000 bp in 100 bp increments. NC denotes the negative control. **(C)** Electrophoresis of restriction enzyme digestion products of *GSTP1* exon 5 PCR amplicons, used to distinguish genotypes based on digestion patterns. PCR = polymerase chain reaction; bp = base pair; GST = glutathione S-transferase; CYP1A1 = cytochrome P450 family 1 subfamily A member 1; NC = negative control.

*GSTM1* and *GSTT1* genes were analyzed with multiplex PCR. All 180 samples were subjected to PCR with the appropriate primers and then analyzed on 2% Agarose gel by electrophoresis. The sizes of the PCR products were: *GSTM1*–215 bp, *GSTT1*–480 bp, and CYP1A1 (control) – 312 bp (Figure 2A).

An internal control for successful PCR amplification was the 312 bp band for CYP1A1 gene which reacted with all the samples. Lanes 33, 38, 40: subjects carrying both *GSTT1* + and *GSTM1* + genotype. Lanes 29, 35: *GSTT1*– and *GSTM1* + individuals. Lanes 30, 32, 34, 37: *GSTT1* + and *GSTM1*– individuals. Lanes 36, 39: deletion of both *GSTM1* and *GSTT1* in individuals.

The results of the multiplex PCR detection were shown in Supplementary Table S1. In the study group, 50 individuals (55.6%) had deletion of the *GSTM1* gene, and in the control group it was present in 45 individuals (50%). 53 (58.9%) individuals in the study group and 45 (50%) in the control group showed deletion of *GSTT1* gene. These findings show that the number of GSTM1 deletions among the residents of the Temirtau region is higher and that there is a significant increase of *GSTT1* gene deletions. This implies an increased risk of aplastic anemia in patients with *GSTM1* and *GSTT1* gene knock outs.

### 3.3 Polymorphism in exon 5 of the GSTP1 gene

All 180 samples were tested for polymorphisms in exon 5 of the *GSTP1* gene by PCR and analyzed by electrophoresis on a 3% agarose gel (Figure 2B). The digestion results of exon 5 of the *GSTP1* gene with restriction enzymes for all 180 samples are presented in Supplementary Table S1.

All 180 samples were analyzed by PCR and restriction for *GSTP1* gene exon 5. The distribution of three observed genotypes is represented in the electropherogram (Figure 2C):

Approximately bands 66, 67, 69 and 70 were homozygous *GSTP1* Ile/Ile genotype.

Bands at 71 and 72 correspond to the heterozygous *GSTP1* genotype (Ile/Val), respectively.

Band 69 homologous to homozygous mutant allele *GSTP1* Val/Val.

Lanes 66, 67, 69, and 70 represent the *GSTP1* Ile/Ile genotype. Lanes 71 and 72 refer to the *GSTP1* Ile/Val genotype. Lane 69 is the mutant *GSTP1* Val/Val genotype. L = DNA size marker (O’GeneRuler^™^ 100 bp Plus).

Table 4 summarizes the frequency distribution of these genotypes. In the Temirtau population the *GSTP1* Ile/Ile genotype was found in 60% of individuals; the *GSTP1* Ile/Val genotype in 35.3%, and the *GSTP1* Val/Val genotype was found in 4.5% of the population. The control group had a better prevalence of Ile/Ile genotype (72.2%) and a worse frequency in Ile/Val genotype (22.2%) compared with the patients. The mutated Val/Val genotype was present in 5.6% of the control group. Table 4 summarizes these frequencies, indicating that *GSTT1*-null and *GSTM1*-null genotypes were significantly higher among residents (*p* < 0.05), suggesting reduced detoxification capacity.

**Table 4:** Alleles and genotypes frequency of *GSTM1*, *GSTT1*, *GSTP1* and *GCLM* genes in individuals from Temirtau region and control group.

Genes	Genotype frequency % (*n*)	
	Residents of Temirtau	Control group
*GCLM*		
*C/C*	93.3 (84)	98.9 (89)
*C/T*	4.4 (4)	1.1 (1)
*T/T*	2.2 (2)	−
*GSTP1*		
*Ile/Ile*	60 (54)	72.2 (65)
*Ile/Val*	35.6 (32)	22.2 (20)
*Val/Val*	4.4 (4)	5.6 (5)
*GSTT1*		
+	41.1 (37)	70 (63)
−	58.9 (53)	30 (27)
*GSTM1*		
+	44.4 (40)	50 (45)
−	55.6 (50)*	50 (45)
*GSTT1 + GSTM1*	12.2 (11)	33.3 (30)

* Shows significant difference with control group. *p* < 0.05. GST = glutathione S-transferase; GCLM = glutamate-cysteine ligase modifier subunit; Ile = isoleucine; Val = valine.

The elevated frequency of the heterozygous Ile/Val genotype among residents of Temirtau as opposed to the control group suggests that there are possible environmental or genetic factors responsible for the differential distribution of *GSTP1* polymorphism in the population of Temirtau.

Associations between behavioral symptoms and genotypes were evaluated. Supplementary Table S2 shows that individuals with combined *GSTM1*-null, *GSTT1*-null, and *GSTP1* Val/Val genotypes reported the highest rates of fatigue and other clinical symptoms, suggesting a high-risk subgroup for Hg toxicity.

### 3.4 Molecular docking findings

Molecular docking analysis revealed that MeHg exhibits low but notable binding affinities with several key epigenetic regulatory proteins, as summarized in Table 5. The binding affinities ranged from −0.9 to −1.1 kcal/mol, indicating weak interactions, yet these may still be biologically relevant due to the high reactivity of MeHg and the sensitivity of epigenetic enzymes to structural perturbations. For comparison under identical parameters, positive control ligands showed substantially stronger affinities: RG108 (a non-nucleoside DNMT1 inhibitor) and trichostatin A (TSA) (a class I/II HDAC inhibitor) docked at ~ − 7.6 to −8.2 kcal/mol, consistent with their established potencies against DNMTs and HDACs in the literature (23–25). Thus, although MeHg exhibits markedly weaker non-covalent docking scores, its high electrophilicity and strong thiophilicity (propensity to bind cysteine/thiol groups) mean that even transient interactions near cysteine- and aromatic-rich regions of epigenetic enzymes could plausibly induce allosteric perturbations.

**Table 5:** The results of the affinity of targeted proteins with MeHg.

Protein	Binding affinity with MeHg (Kcal/mole)
DNMT1	−1.1
HDAC1	−0.9
HDAC2	−1.1
HDAC3	−1.1
HDAC6	−0.9
SIRT1	−1.1

MeHg = MeHg; DNMT1 = DNA (cytosine-5)-Metransferase 1; HDAC = histone deacetylase; SIRT1 = sirtuin 1; kcal/mol = kilocalories per mole.

**Figure 3:**
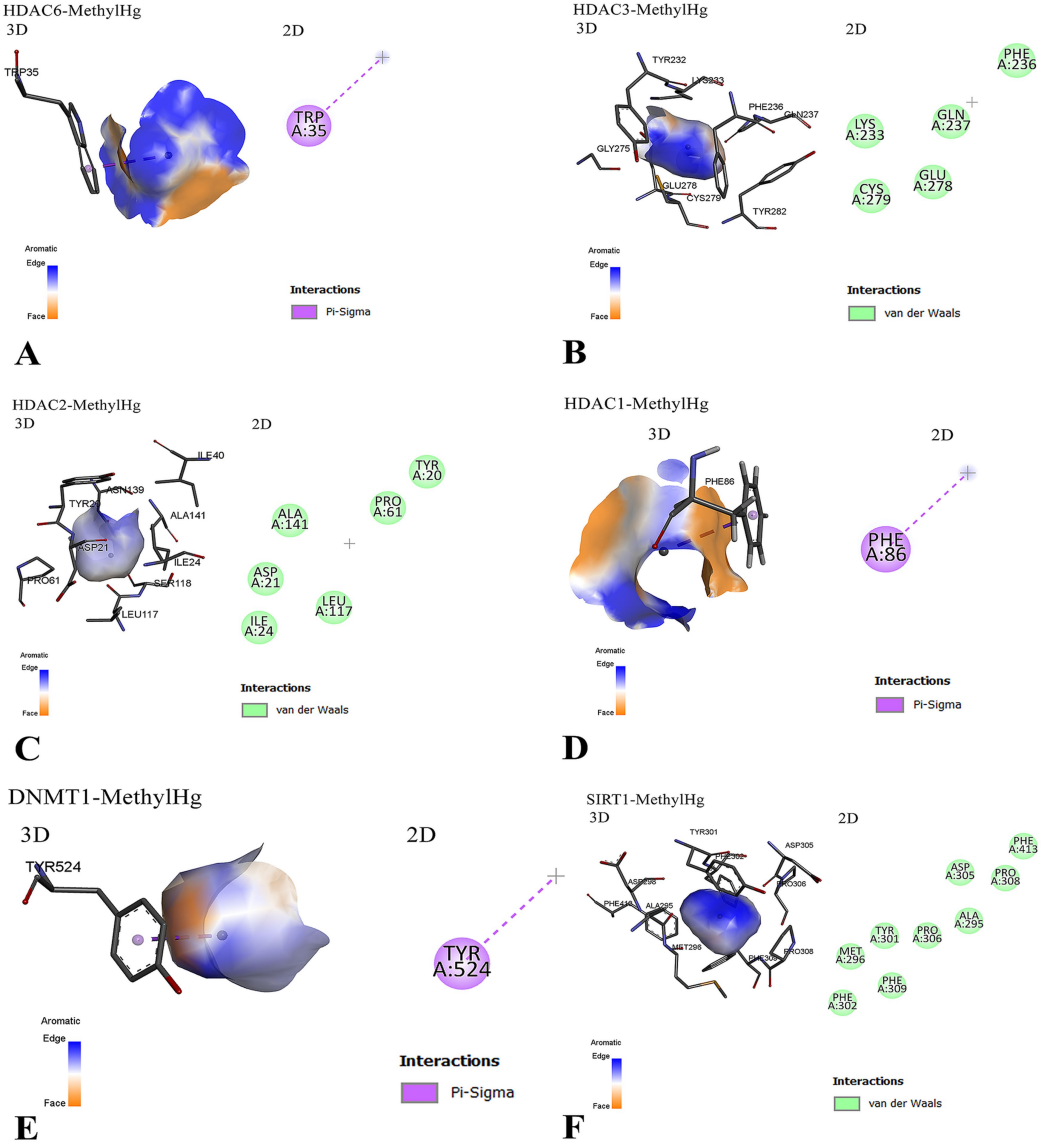
Molecular interactions between MeHg and epigenetic regulatory proteins are illustrated. **(A)** MeHg interacts with HDAC6 via a *π*–*σ* bond involving the aromatic residue tryptophan 35 (TRP35), with the orientation of the aromatic ring facilitating this binding. **(B)** MeHg binds near a cluster of residues in HDAC3, including aromatic (PHE, TYR), polar (GLU, GLN), and sulfur-containing (CYS) amino acids. The interaction is primarily through van der Waals forces, suggesting weak but potentially disruptive binding. Cysteine 279 (CYS279) may play a critical role due to MeHg’s affinity for thiol groups. **(C)** MeHg interacts with HDAC2 predominantly through weak hydrophobic interactions, implying reversible yet potentially functional alterations. **(D)** MeHg targets HDAC1 through a π–σ interaction with phenylalanine 86 (PHE86), which may induce conformational or functional disruptions. **(E)** A similar π–σ interaction is observed between MeHg and tyrosine 524 (TYR524) of DNMT1, suggesting a recurring mode of binding across multiple epigenetic regulators. **(F)** The interaction between MeHg and SIRT1 is shown in both 3D and 2D views. The 3D model (left) highlights the binding pocket and key interacting residues (e.g., TYR301, PHE302, MET296), while the 2D diagram (right) maps van der Waals interactions involving ALA295, PHE302, and ASP305. MeHg = MeHg; DNMT1 = DNA (cytosine-5)-Metransferase 1; HDAC = histone deacetylase; SIRT1 = sirtuin 1; PHE = phenylalanine; TYR = tyrosine; TRP = tryptophan; CYS = cysteine; GLU = glutamic acid; GLN = glutamine; MET = methionine; ALA = alanine; ASP = aspartic acid; vdW = van der Waals.

To further elucidate these interactions, Figure 3 presents a detailed visualization of the molecular interactions between MeHg and the targeted proteins. The docking results highlight specific residues involved in binding, with aromatic *π*–*σ* interactions playing a recurring role. For instance, MeHg binds to HDAC6 via tryptophan 35 (TRP35) and to HDAC1 via phenylalanine 86 (PHE86), while interaction with DNMT1 occurs through tyrosine 524 (TYR524). These interactions, although primarily governed by weak van der Waals forces and hydrophobic contacts, may still disrupt the normal enzymatic activity of these proteins.

Additionally, the interaction with HDAC3 involves proximity to a cluster of chemically diverse residues, including polar (GLU, GLN), aromatic (PHE, TYR), and sulfur-containing (CYS) amino acids, notably CYS279, which is a plausible target due to the high affinity of Hg for thiol groups. Similarly, the binding of MeHg to HDAC2 and SIRT1 also involves non-covalent interactions that may perturb structural or functional properties.

Collectively, these findings suggest that MeHg, despite its low binding energies, may interfere with multiple epigenetic regulators through specific residue interactions, particularly involving aromatic and thiol-containing side chains. This supports a potential mechanism by which MeHg exerts its epigenotoxic effects.

## 4 Discussion

These results reinforce MeHg’s status as an important environmental and human health risk. Our data suggest that this association is driven largely by Hg bioaccumulation resulting from eating fish and shellfish, indicating that dietary intake is an important determinant of Hg exposure in humans. Because MeHg is prevalent in aquatic ecosystems, estimating dietary intake of Hg through fish consumption is important for reducing exposure risks (26). The observed frequencies of *GSTP1* Ile105Val (rs1695) and *GCLM* –588C/T (rs41303970) polymorphisms in Temirtau residents were comparable to those in Central Asian reference populations from the 1,000 Genomes Project, supporting the representativeness of our sample.

The current study is the first to uncover critical insights into the link between Hg exposure and genetic polymorphisms found in the specific population of Temirtau, a region that faces severe industrial Hg pollution. These results showed that there were significant differences in the frequencies of GST polymorphisms; *GSTM1*, *GSTT1*, *GSTP1* and *GCLM* in this population for susceptibility to Hg toxicity. In particular, our results indicate that some genotypes may allow for increased susceptibility to the neurotoxic effects of MeHg, consistent with the literature showing that polymorphisms in GSTs contribute to variations in detoxification (20).

Additionally, previous publications have provided associations between GST gene polymorphisms (27) and environmental pollutants, indicating that individuals with nulls for *GSTM1* and *GSTT1* have impaired detoxification, which will in turn lead to increased Hg accumulation and consequent increased toxicity (12). On the contrary, Our findings show that while the high frequencies of these polymorphisms in the Temirtau population are similar to those observed in other Hg-contaminated regions, the clinical manifestations appear notably less severe than those reported in Minamata disease, likely due to differences in exposure route, duration, and Hg speciation (21). This may indicate that the genetic predisposition plays a crucial role, but the eventual effect of Hg exposure on health can differ considerably depending on other external and lifestyle conditions, such as dietary behaviors including fish intake which represents a significant route of Hg exposure (28).

Our findings from the genetic analysis and molecular docking collectively provide complementary insights into the mechanisms of Hg toxicity in the Temirtau population. The combined data suggest an oxidative-stress–mediated epigenetic pathway, where glutathione depletion caused by GSTM1/GCLM variants may enhance MeHg-induced inhibition of DNMT1 and HDACs, leading to altered DNA-methylation and histone-acetylation patterns. The high prevalence of *GSTM1*-null and *GSTT1*-null genotypes among residents indicates reduced detoxification capacity, consistent with previous reports showing impaired clearance of Hg in individuals lacking these enzymes. Likewise, the increased frequency of *GSTP1* Ile/Val and Val/Val genotypes suggests altered enzymatic activity and reduced conjugation efficiency, which may contribute to higher internal Hg burdens. Together, these polymorphisms define a genetic background of vulnerability to Hg exposure. The molecular docking analysis supports and extends these observations by demonstrating that MeHg interacts directly with epigenetic regulators such as DNMT1, HDAC isoforms, and SIRT1. Although the binding affinities were weak (−0.9 to −1.1 kcal/mol), the interactions involved critical aromatic and cysteine residues within these enzymes, suggesting that even transient binding may disrupt DNA Meation and histone acetylation. These docking outputs should be interpreted as preliminary in silico predictions that require experimental validation. In particular, enzyme activity assays (e.g., DNMT1 Metransferase and HDAC deacetylase assays) and cysteine-blocking/competition experiments would be needed to confirm whether MeHg perturbs catalytic function via thiol-mediated or allosteric mechanisms. We have therefore framed our docking as hypothesis-generating, consistent with best practices for AutoDock Vina workflows (23), and we have contextualized MeHg against well-characterized DNMT1/HDAC inhibitors (e.g., RG108, TSA) to clarify the magnitude gap between canonical ligands and MeHg (24, 25).

Such epigenetic perturbations could amplify the toxic effects of impaired detoxification in genetically susceptible individuals. For example, carriers of *GSTM1*-null and *GSTT1*-null genotypes may accumulate higher Hg levels, which in turn could exacerbate DNMT1 inhibition or HDAC dysregulation, leading to long-term gene expression changes. Taken together, these results highlight a dual mechanism of susceptibility in which genetic polymorphisms reduce the enzymatic clearance of Hg, while epigenetic enzyme interactions alter transcriptional regulation and cellular stress responses. The convergence of these pathways suggests that populations with unfavorable *GSTM1*, *GSTT1*, or *GSTP1* variants may be particularly vulnerable to the subtle epigenotoxic effects of Hg. This integrated interpretation strengthens the biological plausibility of our findings and underscores the importance of combining genetic epidemiology with molecular modeling in environmental health research. The integration of genetic and docking data suggests a synergistic mechanism: GST polymorphisms reduce mercury clearance, while MeHg interactions with DNMT1 and HDACs may amplify epigenetic stress, jointly contributing to interindividual susceptibility.

Furthermore, our molecular docking analysis provides new mechanistic insights into how MeHg interacts with epigenetic regulators and detoxification-related enzymes. The docking results demonstrated that MeHg has a binding affinity with DNMT1, HDAC1, HDAC2, HDAC3, HDAC6, and SIRT1, suggesting a potential role in epigenetic modifications and transcriptional regulation. The relatively low binding energies (ranging from −0.9 to −1.1 kcal/mol) suggest weak, non-covalent interactions; however, such low-affinity bindings can still have biologically significant consequences, particularly when they occur at functionally critical regions of epigenetic enzymes. For example, even transient or weak interactions can allosterically alter protein conformation, interfere with substrate access, or disrupt protein–protein interactions, ultimately leading to impaired enzymatic function such as histone deacetylation or DNA Meation. Notably, these interactions are specific to MeHg, as this compound can form characteristic *π*–*σ* interactions with aromatic residues (e.g., PHE, TYR, TRP) and exhibits a selective affinity for thiol-containing residues (e.g., CYS) despite the low binding energy. This specificity suggests a targeted yet subtle mode of interference by MeHg with the epigenetic regulatory machinery. These findings align with previous research indicating that heavy metals, including Hg, may alter gene expression through epigenetic mechanisms, leading to long-term toxic effects (22).

In particular, the interaction with DNMT1 suggests that MeHg may influence DNA Meation patterns, which could contribute to altered gene expression in detoxification pathways. Similarly, the interactions with HDAC family proteins indicate potential disruptions in histone deacetylation, which is essential for maintaining normal chromatin structure and gene regulation. The interaction with SIRT1, a key regulator of cellular stress responses, further supports the hypothesis that MeHg exposure may induce oxidative stress-related epigenetic modifications, thereby influencing susceptibility to toxicity (29).

In addition, this study is the first to investigate genetic polymorphisms associated with Hg toxicity in this community, advancing our understanding of the relationship between genetic susceptibility and environmental exposure. These dynamics are not only important for informing policy about environmental regulation but also key to designing and implementing targeted public health interventions to meet the needs of people living in polluted environments (30). Our results highlight the importance of monitoring levels of Hg exposure and the need to develop tailored mitigation strategies, especially amongst vulnerable groups such as the population of Temirtau.

## 5 Conclusion

Hg remains a significant public health concern, particularly due to its neurotoxic effects on vulnerable populations. This study highlights the importance of assessing the correlation between Hg exposure and genetic polymorphisms in *GSTM1*, *GSTT1*, *GSTP1*, and *GCLM* among residents of Temirtau, a region with considerable Hg contamination. While exposure levels in this population may not be as extreme as those seen in historical cases like Minamata disease, our findings underscore the need for continuous monitoring of Hg toxicokinetics and toxicodynamics, particularly in underrepresented and industrially affected communities.

Furthermore, our molecular docking analysis provided crucial insights into the interactions between Hg and key detoxification enzymes. The results suggest that specific polymorphic variants of *GSTM1*, *GSTT1*, *GSTP1*, and *GCLM* may alter binding affinities and enzymatic activity, potentially influencing an individual’s ability to detoxify Hg. These findings enhance our understanding of Hg metabolism at a molecular level and reinforce the role of genetic susceptibility in determining health risks associated with heavy metal exposure.

Beyond environmental surveillance, raising public awareness about safe dietary practices and individual genetic susceptibility to Hg toxicity is crucial for mitigating health risks. By integrating molecular insights with epidemiological research and policy-driven interventions, we can develop more effective prevention strategies and improve public health outcomes. Addressing both the biochemical mechanisms of Hg toxicity and the socioeconomic realities of affected communities will be essential in reducing the long-term consequences of industrial pollution and enhancing overall well-being.

## Data Availability

The datasets presented in this study can be found in online repositories. The names of the repository/repositories and accession number(s) can be found in the article/Supplementary material.
