# Supplementary material for: Mercury exposure, epigenetic modifications, and genetic susceptibility: insights from molecular docking and population analysis
Source: Front Public Health. 2025 Nov 17;13:1710032. doi: 10.3389/fpubh.2025.1710032 (PMC12665739; doi:10.3389/fpubh.2025.1710032)
Supplement: Supplementary file 1 [file Data_Sheet_1.docx]

Mercury exposure, epigenetic modifications, and genetic susceptibility: insights from molecular docking and population analysis

Bakhtiyar Serik ^1^, Lyazzat E. Shinetova ^2^, Natalya V. Efimova ^3^, Saulemay A. Bekeeva ^4^, Balkiya M. Abdrakhmanova ^5^, Aliya O. Dauletova ^4^, Roza K. Suleimenova ^4^, Nadiar M. Mussin ^6^, Afshin Zare ^7^, Ramazon Safarzoda Sharoffidin ^8^, Amin Tamadon ^9,^*

^1^ Medical School, M. Kozybayev North Kazakhstan University, Petropavlovsk, Kazakhstan; [bakhtiyarserik@gmail.com](mailto:bakhtiyarserik@gmail.com) (B.S.)

^2^ Psychometric Laboratory, National Testing Center, Astana, Kazakhstan; [lyazzat_daniar@mail.ru](mailto:lyazzat_daniar@mail.ru) (L.E.S.)

^3^ East Siberian Institute of Medical and Ecological Research, Angarsk, Russia; [medecolab@inbox.ru](mailto:medecolab@inbox.ru) (N.V.E.)

^4^ Department of Hunting and Fisheries, Kazakh Agro-Technical University named after S. Seifullin, Astana, Kazakhstan; [alima77764@mail.ru](mailto:alima77764@mail.ru) (S.A.B.); [adaulet2506@gmail.com](mailto:adaulet2506@gmail.com) (A.O.D.); [rozasuleimenova@mail.ru](mailto:rozasuleimenova@mail.ru) (R.K.S.)

^5^ LN Gumilyov Eurasian National University, Astana, Kazakhstan; [moldir-arujan@mail.ru](mailto:moldir-arujan@mail.ru) (B.M.A.)

^6^ Department of General Surgery, West Kazakhstan Marat Ospanov Medical University, Aktobe, Kazakhstan; [nadiar_musin@zkmu.kz](mailto:nadiar_musin@zkmu.kz) (N.M.M.)

^7^ International PhD Program in Medicine, College of Medicine, Taipei Medical University, Taipei, Taiwan; [d142114028@tmu.edu.tw](mailto:d142114028@tmu.edu.tw) (A.Z.)

^8^ Department of Pharmaceutical Technology, Avicenna Tajik State Medical University, Dushanbe, Tajikistan; [safarzoda.r.90@gmail.com](mailto:safarzoda.r.90@gmail.com) (R.S.S.)

^9^ Department of Natural Sciences, West Kazakhstan Marat Ospanov Medical University, Aktobe, Kazakhstan; [amintamaddon@yahoo.com](mailto:amintamaddon@yahoo.com) (A.T.)

***** Correspondence: [amintamaddon@yahoo.com](mailto:amintamaddon@yahoo.com); Tel: +7 705 629 9350

Table S1. Multiplex PCR results using the GSTM1 and GSTT1 genes

| Item no.* | GSTM1 215 bp | GSTT1 480 bp | GSTP1 |
| --- | --- | --- | --- |
| 1 | + | + | Homozygote |
| 2 | - | + | Heterozygote |
| 3 | - | - | Heterozygote |
| 4 | - | - | Homozygote |
| 5 | - | + | Mutant |
| 6 | - | + | Homozygote |
| 7 | + | + | Homozygote |
| 8 | - | + | Homozygote |
| 9 | + | + | Heterozygote |
| 10 | - | + | Homozygote |
| 11 | - | + | Homozygote |
| 12 | - | + | Homozygote |
| 13 | + | + | Heterozygote |
| 14 | + | - | Heterozygote |
| 15 | + | - | Heterozygote |
| 16 | - | + | Homozygote |
| 17 | - | - | Homozygote |
| 18 | - | - | Homozygote |
| 19 | + | - | Homozygote |
| 20 | + | + | Heterozygote |
| 21 | + | - | Heterozygote |
| 22 | - | - | Heterozygote |
| 23 | + | - | Homozygote |
| 24 | - | - | Mutant |
| 25 | + | - | Heterozygote |
| 26 | - | - | Heterozygote |
| 27 | - | + | Homozygote |
| 28 | + | - | Mutant |
| 29 | + | - | Homozygote |
| 30 | - | + | Homozygote |
| 31 | - | - | Heterozygote |
| 32 | - | + | Homozygote |
| 33 | + | + | Heterozygote |
| 34 | - | + | Homozygote |
| 35 | + | - | Homozygote |
| 36 | - | - | Heterozygote |
| 37 | - | + | Homozygote |
| 38 | + | + | Heterozygote |
| 39 | - | - | Heterozygote |
| 40 | + | + | Homozygote |
| 41 | + | + | Heterozygote |
| 42 | + | - | Homozygote |
| 43 | + | + | Heterozygote |
| 44 | + | + | Homozygote |
| 45 | - | + | Homozygote |
| 46 | - | + | Heterozygote |
| 47 | + | + | Heterozygote |
| 48 | + | - | - |
| 49 | + | + | Homozygote |
| 50 | + | + | Homozygote |
| 51 | + | + | Heterozygote |
| 52 | + | - | Homozygote |
| 53 | + | + | Homozygote |
| 54 | + | + | Heterozygote |
| 55 | - | + | Heterozygote |
| 56 | + | + | Homozygote |
| 57 | - | + | Homozygote |
| 58 | + | + | Heterozygote |
| 59 | - | + | Homozygote |
| 60 | - | + | Homozygote |
| 61 | - | - | - |
| 62 | - | + | Homozygote |
| 63 | + | + | Homozygote |
| 64 | + | + | Heterozygote |
| 65 | + | + | Homozygote |
| 66 | + | + | Homozygote |
| 67 | + | - | Homozygote |
| 68 | + | + | Mutant |
| 69 | + | + | Homozygote |
| 70 | - | - | Homozygote |
| 71 | + | + | Heterozygote |
| 72 | - | - | Heterozygote |
| 73 | - | + | Homozygote |
| 74 | + | - | Homozygote |
| 75 | + | - | Heterozygote |
| 76 | + | - | Homozygote |
| 77 | + | - | Heterozygote |
| 78 | - | + | Homozygote |
| 79 | + | + | Heterozygote |
| 80 | - | - | Homozygote |
| 81 | + | - | Homozygote |
| 82 | - | - | Homozygote |
| 83 | - | + | Homozygote |
| 84 | + | - | Heterozygote |
| 85 | - | - | Homozygote |
| 86 | + | - | Homozygote |
| 87 | + | + | Homozygote |
| 88 | + | + | Homozygote |
| 89 | - | + | Heterozygote |
| 90 | + | + | Homozygote |
| 91 | - | + | Heterozygote |
| 92 | - | + | Heterozygote |
| 93 | - | + | Homozygote |
| 94 | - | - | Heterozygote |
| 95 | - | - | Heterozygote |
| 96 | + | - | Heterozygote |
| 97 | + | - | Heterozygote |
| 98 | - | + | Homozygote |
| 99 | - | + | Homozygote |
| 100 | - | - | Homozygote |
| 101 | - | + | Mutant |
| 102 | + | - | Homozygote |
| 103 | + | + | Heterozygote |
| 104 | - | - | Heterozygote |
| 105 | + | - | Homozygote |
| 106 | - | - | Homozygote |
| 107 | + | - | Homozygote |
| 108 | - | + | Homozygote |
| 109 | + | + | Homozygote |
| 110 | - | - | Homozygote |
| 111 | - | + | Heterozygote |
| 112 | - | + | Homozygote |
| 113 | - | + | Heterozygote |
| 114 | - | + | Homozygote |
| 115 | + | - | - |
| 116 | - | - | Homozygote |
| 117 | - | + | Homozygote |
| 118 | - | - | Homozygote |
| 119 | - | + | Homozygote |
| 120 | - | + | Homozygote |
| 121 | + | - | Homozygote |
| 122 | - | - | Homozygote |
| 123 | - | - | Homozygote |
| 124 | - | - | Homozygote |
| 125 | - | - | - |
| 126 | - | - | - |
| 127 | - | - | - |
| 128 | + | - | Homozygote |
| 129 | - | - | Mutant |
| 130 | + | - | Homozygote |
| 131 | + | - | Heterozygote |
| 132 | - | - | Homozygote |
| 133 | - | + | Homozygote |
| 134 | - | - | Homozygote |
| 135 | + | + | Homozygote |
| 136 | - | - | Homozygote |
| 137 | - | - | Homozygote |
| 138 | + | + | Homozygote |
| 139 | + | - | Heterozygote |
| 140 | - | + | Homozygote |
| 141 | - | - | Homozygote |
| 142 | - | - | Homozygote |
| 143 | - | - | Homozygote |
| 144 | - | + | Homozygote |
| 145 | + | + | Homozygote |
| 146 | - | + | Mutant |
| 147 | - | + | Mutant |
| 148 | - | - | Homozygote |
| 149 | + | - | Homozygote |
| 150 | + | + | Homozygote |
| 151 | - | + | Homozygote |
| 152 | - | + | Homozygote |
| 153 | + | + | Heterozygote |
| 154 | + | + | Homozygote |
| 155 | + | - | Heterozygote |
| 156 | - | - | Homozygote |
| 157 | + | + | Homozygote |
| 158 | - | + | Homozygote |
| 159 | + | + | Homozygote |
| 160 | - | + | Homozygote |
| 161 | - | + | Homozygote |
| 162 | - | + | Homozygote |
| 163 | - | + | Homozygote |
| 164 | + | + | Heterozygote |
| 165 | - | - | Heterozygote |
| 166 | + | - | Homozygote |
| 167 | + | - | Homozygote |
| 168 | - | - | Heterozygote |
| 169 | - | - | Homozygote |
| 170 | + | - | Homozygote |
| 171 | - | + | Homozygote |
| 172 | - | + | Heterozygote |
| 173 | - | - | Heterozygote |
| 174 | - | + | Homozygote |
| 175 | - | + | Homozygote |
| 176 | - | + | Mutant |
| 177 | - | + | Heterozygote |
| 178 | - | - | Homozygote |
| 179 | - | + | Homozygote |
| 180 | - | + | Homozygote |

Abbreviations: PCR = polymerase chain reaction; GST = glutathione S-transferase; CYP1A1 = cytochrome P450 family 1 subfamily A member 1; bp = base pair.

Table S2. Comparison of demographic, lifestyle, clinical, and genetic profiles between temirtau residents and matched control group

| Variable | Temirtau Residents (n=90) | Controls (n=90) | Notes |
| --- | --- | --- | --- |
| Age (mean ± SD) | 64.2 ± 10.5 years | 59.6 ± 12.3 years | Controls slightly younger; both groups predominantly middle-aged/elderly |
| Sex (Female:Male) | 52:38 | 54:36 | Comparable gender distribution |
| Nationality | 48 Kazakh, 42 Russian | 90 Kazakh | Controls exclusively Kazakh; Temirtau group ethnically mixed |
| Residence Duration | >20 years (all) | >15 years (all) | Both groups are long-term residents |
| Smoking Status | 12 current smokers | 0 (excluded) | Smoking was an exclusion criterion for controls |
| Alcohol Use | 24 (26.7%) report moderate/regular use | 0 (excluded) | Alcohol consumption not allowed in controls |
| Chronic Diseases | 90% with hypertension | 6 with АГ (excluded) | High comorbidity in exposed group; excluded from controls |
| Fatigue Complaints | 41 (45.5%) report chronic fatigue | 3 (minor, not chronic) | Significant difference in fatigue prevalence |
| Chest Pain/Cardiac | 35 (39%) with stabbing chest pain | 0 | Symptom not reported in controls |
| Urinary Mercury (µg/L) | 1.8 ± 1.2 | 0.4 ± 0.3 | Statistically higher exposure in Temirtau group |
| Genotype (GSTP1) | 56 Ile/Ile, 21 Ile/Val, 13 Val/Val | 53 Ile/Ile, 17 Ile/Val, 2 Val/Val | Higher frequency of Val/Val in Temirtau group |
| Genotype (GSTM1) | 48 null, 42 present | 27 null, 63 present | GSTM1-null significantly more frequent in exposed group |
| Genotype (GSTT1) | 39 null, 51 present | 22 null, 68 present | Similar trend as GSTM1 |
| Combined GST-null | 21 participants with both GSTM1 & GSTT1 null | 6 participants | Risk group for poor detoxification |
| Val/Val + GST-null | 9 participants with Val/Val & both null | 0 | High-risk cluster for toxicity symptoms |
| Occupational Hazards | 28 miners, drivers, welders | 0 (excluded) | Controls excluded from industrial or chemical exposure |
| Family History Study | 5 families studied; 3 showed shared genotypes and symptoms | Not performed | Familial pattern supports genetic susceptibility |

Abbreviations: GST = glutathione S-transferase; Val = valine; Ile = isoleucine; µg/L = micrograms per liter.
